# Can Trust Bring Satisfaction to the Festival Under Pandemic?

**DOI:** 10.1177/21582440221147248

**Published:** 2023-01-09

**Authors:** Jiamin Liu, Eusebio Chiahsin Leou, Chaozhan Chen, Xi Li

**Affiliations:** 1 Chengdu University of Information Technology, China; 2 City University of Macau, Taipa, China

**Keywords:** COVID-19 pandemic, festivalscape, trust, experience quality, satisfaction, Macau Light Festival

## Abstract

In existing festival research, trust is often placed at the post-experience stage by scholars, and there is no research to explore the relationship between festivalscape, trust, and experience. In addition, the main function of trust is to reduce uncertainty and risk perception. Existing festival-related research scenarios do not have typical risks, which will limit the findings of the research. This research focuses on festivals under covid-19 pandemic, which is a typical risky scenario. Exploring the antecedent variables and the consequences of the trust of the festival participants in this context may therefore make up the deficiency of existing research. Several interesting findings have been made: the perception of the festivalscape during the pandemic has been significantly simplified. Epidemic prevention measures and staff may increase trust, but trust cannot have a direct and significant impact on festival satisfaction. While staff positively strengthen trust, they will have a significant negative impact on satisfaction.

## Introduction

In contrast to previous crises and pandemics, COVID-19 has had a profound and long-lasting effect on socioeconomic activities and tourism industries, ultimately resulting in global economic recession and depression (Sigala, 2020). Given the uncertainty and anxiety associated with COVID-19 and consumers’ fears of engaging in leisure and economic activities that have followed lockdowns, establishing trust is critical for increasing purchase intentions (Kim & Liu, 2022). Scholars have emphasized the importance of trust as a marketing tool in times of crisis, as trust has the potential to reduce uncertainty and perceived risk (Chen & Barnes, 2007). Additionally, trust is a critical component that binds a society together and supports people’s attitudes and behaviors (Fancourt et al., 2020).

The importance of trust in research on health management has been emphasized numerous times in the context of the pandemic. Scholars have discovered that the effectiveness of COVID-19 measures and the appropriateness of communications influence public trust in health authorities (Holroyd et al., 2020). Additionally, studies have demonstrated that public trust in COVID-19 prevention measures is influenced by demographic factors. Thus, older and more educated women place a higher premium on these measures than other demographics do (Almutairi et al., 2020). Scholars have also discussed the impacts of news (Bratu, 2020a, 2020b; Dobson-Lohman & Potcovaru, 2020) and social media (Clark, 2020) on people’s cognitive factors during the pandemic from a communication science perspective, focusing on the psychological factors that affect individuals (Lăzăroiu et al., 2020). Additionally, recent tourism research has emphasized the importance of tourist trust for alleviating negative emotions, such as threats and fears (Shin et al., 2022; Zheng et al., 2021a) also focused on the effect of trust on leisure obstacles, while other scholars have evaluated social distancing. Concerning the impacts of consumer trust, the maintenance of social distancing at hotels during the pandemic is a more favorable measure for tourists’ perceptions than promotion is (Kim & Liu, 2022). That is, social distancing can be considered a form of perceivable behavior control that can help reduce risk perception. Various contexts of the COVID-19 pandemic thus demonstrate that trust is critical to the tourism industry’s recovery.

According to Deutsch (1962), a psychologist, trust is an irrational choice made by individuals when they are confronted with uncertain situations, and it is determined by the psychology and behavior of individuals when they respond to specific situations. A scene thus serves as the foundation for consumers to develop trusting behaviors. However, research on tourism activities in the context of a pandemic is limited. Regarding festivals, the term “festivalscape” encompasses a physical environment that comprises both tangible and intangible elements (Mason & Paggiaro, 2012). Today, festivals and tourism activities should therefore not only provide participants with experiences and backgrounds but also implement appropriate pandemic prevention measures to establish a novel festivalscape. Confronted with the new challenges posed by the pandemic’s impacts on the tourism industry, some scholars have increasingly focused on exploration. Other scholars have emphasized the importance of creating a safe environment to help tourists engage in beneficial behaviors and build trust (e.g., allowing tourists to maintain social distance) (Shin et al., 2022).

Previous research has established that the quality of a consumer experience has a significant impact on trust (Assaker et al., 2020; Wu et al., 2018). Few scholars, however, have examined the effect of trust on consumer confidence, although Rios and Riquelme (2010) have emphasized the important role of trust in the relationship between consumers and products. Consumers who trust a brand are more likely to engage in brand activities, enhancing brand equity, which includes brand connection, perceived quality, and brand loyalty (Chatzipanagiotou et al., 2016; Fatma et al., 2015). Additionally, Biedenbach et al. (2019) established the beneficial effect of trust on perceived quality in a B2B context, but this area has received little attention in tourism research.

Accordingly, this study analyses festivalscapes in the context of COVID-19 to address the following questions: Does a festivalscape have a significant positive effect on participant trust? How does a festivalscape affect participant experience quality? How does a festivalscape affect participant experience quality in the context of the COVID-19 pandemic? Finally, does a participant’s trust significantly improve the quality of the participant’s experience?

## Literature Review and Hypothesis Development

### Festivalscape

Bitner (1992) coined the term “servicescape” for the service industry, which comprises various environmental elements that result from the careful design and control of service locations, including atmospheres, spatial layouts, and functions, for example, signs, graphics, and artifacts. On this basis, Lee et al. (2008) created the term “festivalscape” to apply this concept in the study of festivals. According to environmental psychologists, when an environment changes, personal behavior changes as well (Gifford, 2007). Scholars have conducted research on festivalscapes with this perspective in mind.

Yang et al. (2011) found that festivalscapes, including both tangible service facilities and intangible services and projects, are dynamic atmospheres that participants encounter. A festivalscape is also commonly understood to be a physical environment containing tangible factors and a festive atmosphere (Mason & Paggiaro, 2012). Scholars previously addressed festivalscapes as multidimensional variables, comprising between three and eight dimensions. For example, Yang et al. (2011) and Choe et al. (2018) both divided a festivalscape into five dimensions. Bruwer (2014) and Yoon et al. (2010) separated a festivalscape into six dimensions, and Mason and Paggiaro (2012) proposed a three-dimensional festivalscape. Moreover, Selmi et al. (2021) identified four dimensions of a festivalscape, that is, project, atmosphere, information, and employees, which combine to create a festival environment. The concept of a festivalscape with the most diverse connotations was proposed by Lee et al. (2008) and includes eight dimensions. Below, Table 1 illustrates the compositions of specific dimensions.

**Table 1.** The Internal Dimensions of Festivalscape.

Authors	Number of dimensions	Composition of specific dimensions
Mason and Paggiaro (2012)	3	Fun; Comfort; Food.
Yang et al. (2011)	5	Environmental conditions; Space layout; Esthetic atmosphere; Employee services; Entertainment programs.
Choe et al. (2018)	5	Festival venue quality; Festival expense; Festival entertainment and festival programs; Festival logistics; The assistance of festival staff.
Bruwer (2014)	5	Festival characteristics; Festival staff; Entertainment and catering; Amenities for comfort and convenience; Festival venues; Information.
Yoon et al. (2010)	6	Program content; Staff; Details; Facilities; Souvenirs; Convenience.
Lee et al. (2008)	8	Quality of the festival activity plan; The quality of service of staff/ volunteers; The quality and availability of auxiliary services; The quality of food; Souvenirs; Comfort; Accessibility; Information availability.

Although the elements of a festivalscape differ among various authors, the participants at a festival perceive it from a multitude of angles.

### Festivals During the Pandemic

Numerous festivals that were impacted by the COVID-19 pandemic have been canceled or postponed (Jamal & Budke, 2020). Some nations and territories, however, overcame difficulties and held festivals. Because public guidelines and rules enable participants to build a sense of confidence (Anania & Nisticò, 2004; Oksanen et al., 2020; Poortinga & Pidgeon, 2003), various guidelines on crowd gathering and festival management have been provided by many nations and local governments. For example, Beijing and Shanghai in Mainland China have issued guidelines for the management of conventions and exhibitions, and Macau has issued similar guidelines. These guidelines have provided new implications to festival management and have changed festivalscape perceptions. Accordingly, in this research, we analyze the content of specific pandemic prevention guidelines issued by the governments of Beijing, Shanghai, Guangzhou, and Shenzhen, which are China’s major event destinations. We also evaluate the guidelines composed by event organizers for eight events including the Shanghai Tourism Festival 2020, Qingdao Beer Festival 2020, Wuhan Beer Festival 2020, Macau Food Festival, Guangzhou Construction Expo 2020, Hangzhou Strawberry Music Festival 2020, Shenzhen Gift Show, and Shanghai International Import Expo 2020. Through content analysis, this study found that nine activities for pandemic prevention were listed more than three times: measuring human body temperature, supplying personal disinfection supplies, restricting human flow, social distancing, regularly disinfecting, installing pandemic warning signs, separating entrances and exits, implementing closed management, and monitoring participant flow. Table 2 illustrates the compositions of specific measures

**Table 2.** Epidemic Prevention Measures in Event Project Guidelines and Urban Epidemic Guidelines.

Measures	Frequency
1. Measuring human body temperature	11
2. Provide personal disinfection supplies	10
3. Limit the flow of people	9
4. Maintaining social distance	9
5. Regular disinfection	8
6. Installation of epidemic warning signs	4
7. Separate entrances and exits	5
8. Implementation of closed management	4
9. Monitoring the participant flow	3

### Trust

Trust studies can be traced back to psychological research in the 1950s. Trust has attracted the attention of tourism and hospitality scholars since the 1990s (Lewis & Weigert, 1985). Recently, the number of studies on “trust” in the tourism and hospitality industry has risen steadily (Wang et al., 2014). In general, trust refers to the confidence that one party has in the fairness and dignity of the other party involved in a transaction (Crosby et al., 1990) or one partner’s general expectations regarding his or her trading partner’s future performance (Lee & Back, 2008). In short, trust is whether a person believes he or she can depend on others (Pavlou, 2003). Trust will thus reduce customers’ vulnerability in the decision-making phase when customers are in an unpredictable environment (Pavlou et al., 2007). Therefore, trust is a common element in research in the fields of relationship marketing, organizational behavior, sociology, and psychology (Morgan & Hunt, 1994). Many festival scholars have shown the value of trust from the perspectives of brand equity (Lee & Back, 2008), festival support (Song et al., 2014), and participant action (Akhoondnejad, 2016). In their review of tourism trust research, Williams and Baláž (2021) noted that trust is a critical concept in episodic politics, health policies, economic events, and natural disaster environments. When social and natural environments change abruptly, trust changes as well (Williams & Baláž, 2021). Since the COVID-19 pandemic has increased environmental and organizational instability and risks, trust has become more critical than ever. Regarding the sources of trust, studies by scholars in different fields have shown that an essential trust source is one’s current setting (Lee & Teo, 2005; Perrini et al., 2010; Sparks & Browning, 2011). Thus, Harris and Goode (2010) have verified that in an online service environment, the servicescape has a major effect on trust, as servicescapes are critical for establishing consumer trust in online services (Tran & Strutton, 2020). Folgado-Fernández et al. (2021) noted in their study on event management that structural elements in gastronomic and religious events facilitate the formation of emotional engagement among participants. Chen and Chang (2012) have also asserted that lowering perceived risk promotes consumer trust in green products. Accordingly, we speculate that adding pandemic prevention measures to a servicescape will help reduce participants’ perceptions of risk and increase their trust during a pandemic.

This study therefore proposes the following hypothesis:

H1: A festivalscape has a significant effect on trust.

### Experience Quality

In tourism, experience quality refers to the psychological outcomes for visitors who engage in tourism activities (Chen & Chen, 2010). Experience quality can also be conceptualized as tourists’ emotional reactions to their desired sociopsychological benefits (Lian Chan & Baum, 2007). One’s evaluation of experience quality is generally a comprehensive and subjective assessment that emphasizes individual (internal) feelings (Wu et al., 2018). Moreover, Jin et al. (2015) indicated that experience quality involves an assessment of the qualities of interactions, physical environments, and outcomes.

Some researchers have pointed out that factors such as product category, engagement, product complexity and hedonism should be taken into account when evaluating experience quality (Fedler & Ditton, 1986; Wu & Li, 2017). Lemke et al. (2011) suggested that many variables influence the quality of an experience, including other consumers, physical climates, interactions, and supplier processes. A servicescape has been found to influence experience quality in theme parks (Wu et al., 2018), restaurants (Liu & Jang, 2009), resorts (Ismail, 2011), and airports (Choi & Kim, 2015). For instance, Tran et al. (2020) showed that the interpretation of a cafe servicescape by customers directly affects their experience quality. Loureiro (2017) also demonstrated that servicescape elements are effective predictors of medical tourists’ perceived quality. Finally, Agnihotri and Chaturvedi (2018) asserted that a servicescape is a determining factor for restaurant patrons’ perceived quality.

This study therefore proposes the following hypothesis:

H2: A festivalscape affects one’s festival experience quality.

### Experience Quality, Trust, and Satisfaction

Satisfaction is generally defined as a consumer’s overall assessment of a customer experience (Kim et al., 2011). Satisfaction with a festival entails “the sum of participants’ experiences during the festival” (McDowall, 2011). Yoon et al. (2010) described festival satisfaction as one’s overall value of a combination of quality dimensions. Scholars have carried out much empirical research on the role of festival satisfaction, and Grappi and Montanari (2011) have observed that satisfaction is a significant factor for maintaining long-term relationships with participants.

The relationship between festivalscape and satisfaction has been explored by scholars in various contexts. For example, Mason and Paggiaro (2012) have confirmed that there is a strong positive connection between festivalscape and participant satisfaction in the Italian Food and Wine Festival. This result was confirmed in a study of the Macau Food and Wine Festival 2018 (Choe et al., 2018). Beckman et al. (2020) have also established that in the context of food festivals, an eventscape has a significant positive effect on participant satisfaction. Troisi et al. (2019) examined the relationship between festivalscape and satisfaction from a service logic perspective, discovering that festivalscape attributes have a significant positive effect on participant satisfaction. However, the abovementioned research focused exclusively on wine festivals; there is no research on festivalscapes and on satisfaction with different forms of festivals. Due to the broad differences in the festivalscapes of differently themed festivals, investigating art festivals through a case study will help verify the relationship described above.

Quality, satisfaction, and trust have long been considered key principles to explain consumer behaviors after purchases. Researchers have typically agreed that these variables influence intention, retention, and loyalty, which are beneficial to an organization (Bowen & Chen, 2001; Han & Jeong, 2013; Han & Ryu, 2007). Satisfaction is based on the overall experience of customers with quality and information (Yoon et al., 2010). Moreover, Lee et al. (2008) have indicated that festival quality is a key factor affecting participant satisfaction; specifically, the quality of a service experience is positively affects consumer satisfaction (Cronin & Taylor, 1992). Several scholars have also examined the relationship between experience quality and satisfaction in the context of creative tourism, and their findings indicate that the quality of a tourist experience directly affects satisfaction level (Suhartanto et al., 2020). These conclusions were confirmed in Ghorbanzadeh et al.’s (2021) study on Black tourism.

The effect of trust on satisfaction has also been verified in various disciplines. For example, Lee, Song et al. (2013) and Lee, Jan et al. (2013) revealed that employees’ trust in their organization greatly affects their job satisfaction. Tourism studies have shown that trust has a significant impact on experience quality (Bhaduri, 2011) and satisfaction (Chang, 2014; Lee, Jan et al., 2013; Lee, Song et al., 2013). In the field of e-commerce, Ratnasingham (1998) pointed out that trust has a significant impact on customer satisfaction. Moreover, in the field of consumer behavior, Uzir et al. (2021) established that trust in a door-to-door service has a significant positive effect on customer satisfaction. Finally, Al-Ansi et al. (2019) discovered that trust has a significant positive effect on halal customer satisfaction in the hospitality industry, indicating that trust is a key factor that affects customer satisfaction (Pavlou, 2003).

Based on the abovementioned literature, this study proposes the following hypotheses:

H3: A festivalscape has a positive effect on satisfaction.
H4: Trust has a significant effect on festival participant satisfaction.
H5: Festival experience quality has a significant effect on festival participant satisfaction.

### Trust and Experience Quality

Many scholars have pointed out that trust allows decision-makers to reduce their concerns about ambiguous or uncertain information sources (De Jong et al., 2016). Thus, a lack of trust has an effect on customers’ positive quality perception (Maltz & Kohli, 1996). However, studies by other scholars, such as Heisey (1990) and Bhattacherjee (2002), have shown that consumer trust in companies affects the perceived quality of products, which in turn affects consumer purchase intention. Bhaduri’s (2011) research has also shown that trust level has a direct effect on customer perception of product quality. With the development of trust in relationships between customers and suppliers, both parties become more likely to share sensitive information and engage in constructive and equally beneficial creative dialog (Selnes & Sallis, 2003). Thus, Chatzipanagiotou et al. (2016) made the following observation: “Trust facilitates the formation of brand relationships, thereby fostering the growth of brand equity.” In other words, this type of trusting relationship can result in increased perceived quality, brand association, and brand loyalty (Biedenbach et al., 2019). Finally, the uncertainty and risk that COVID-19 engenders have increased participants’ trust expectations. Once this novel level of trust is established, it thus enables participants to perceive a more desirable level of experience.

This study therefore proposes the following hypothesis:

H6: Trust has a significant effect on festival participant experience quality.

### The Mediating Role of Trust and Experience Quality

**Figure 1.**
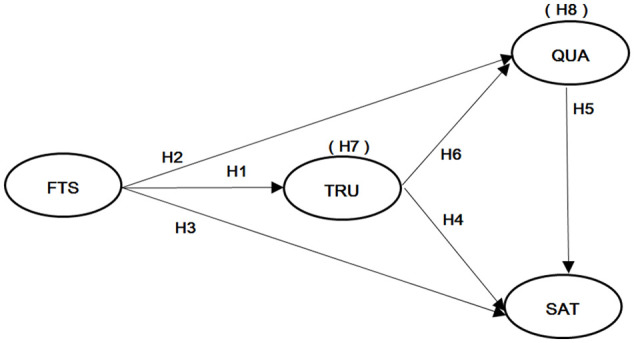
Theoretical Framework. *Note*. FTS = festivalscape; TRU = trust; QUA = experience quality; SAT = satisfaction.

Some scholars have pointed out that in addition to the direct effects of service evaluation, i.e., perceived quality, value and satisfaction (Kamakura et al., 2003), scholars should also pay attention to the role of a relationship framework, for example, trust and relationship commitment, because they are potential mediators of the effect of service evaluation on customer patronage behavior. Cue utilization theory has been applied in tourism studies to show that consumers regard product information, such as price, color, and salesperson attitude, as cues. Consumers will use such cues to evaluate and judge products (Cox, 1962). Thus, when a product conveys positive information, consumers develop a more favorable relationship, for example, based on trust, with it and then have a more favorable opinion of it (Li et al., 2015). Moreover, Tran and Strutton (2020) have revealed that trust acts as a mediator between e-servicescape and behavioral intention in online consumer behavior. Cole and Scott (2004) have also evaluated the mediating role of experience quality between service quality and overall satisfaction. In the context of cultural heritage tourism, Domínguez-Quintero et al. (2020) investigated the mediating role of experience quality between authenticity and satisfaction. In other interdisciplinary fields, DeWitt et al. (2008) have identified trust and emotion as key intermediaries in the relationship between perceived fairness and customer loyalty. Li and Yeh (2010) also examined the relationships between brand awareness, perceived quality, brand loyalty and consumer purchasing intention, testing the mediating role of perceived quality between brand recognition and brand loyalty. Finally, according to Ha and Jang (2012), perceived quality acts as a mediator between restaurant atmosphere and behavioral intention. Accordingly, following the abovementioned papers, this study proposes the following hypotheses:

H7: Trust plays a mediating role between festivalscape and festival experience quality.
H8: Festival experience quality plays a mediating role between festivalscape and festival participant satisfaction.
The theoretical model proposed in this research is depicted in Figure 1.

## Methodology

### Research site

As a tourist destination, Macau has an economy that has deteriorated since the initial outbreak of COVID-19. According to data from the Macau Gaming Inspection and Coordination Bureau, gross gaming revenue in the first 7 months of 2020 amounted to $35.06 billion in Hong Kong dollars, down 79.8% from the previous year. Macau received approximately 3.27 million inbound visitors in the first half of 2020, a sharp decline of 83.9%. Through its increasing control of the COVID-19 pandemic, the Macau SAR government currently hopes to use festivals to boost the local economy. As of late September 2020, Mainland Chinese residents were able to travel to Macau once again. Macau thus immediately launched a series of events, including the “Grand Prix,” the Macau Food Festival, and the Macau Light Festival. Of these large-scale festivals, the Macau Light Festival was the first to be held since the COVID-19 pandemic had begun. This festival was previously held in the month of December but in 2020 it was moved to September to better attract and welcome tourists to Macau.

This festival is inextricably linked to Macau’s world cultural heritage and uses light sculpture performances and lighting installations to create an all-weather attraction that integrates modern technology and art. Since 2014, the festival has been held five times and has been extremely well received by both visitors and locals. Unlike previous festivals, in 2020, numerous anti-pandemic measures were implemented onsite to ensure that attendees and visitors could enjoy the festival’s charm in a healthy and safe environment. Accordingly, the subjects of this study are the attendees of the 2020 Macau Light Festival.

### Measurement

To measure the aspects of the case festivalscape, we relied on existing research findings and integrated the abovementioned pandemic prevention initiatives. We primarily followed Yang et al.’s (2011) festivalscape scale, which is divided into ambient conditions, spatial layouts, facility esthetics, staff resources, and entertainment programs and includes 14 items. This study also used the results of a content analysis of the pandemic prevention guidelines to supplement the scale and measure festivalscape perception (a total of nine items).

The assessment of trust is based on Lee et al.’s (2019) research, which divides trust into five items and three dimensions, that is, perceived comprehensibility, perceived technological competence, and perceived reliability.

Experience quality is assessed by Domínguez-Quintero et al.’s (2020) five items, while satisfaction level is primarily based on the satisfaction scale of Lee et al. (2012), which includes five items. Finally, the questions listed above were adapted to the characteristics of festivals during the COVID-19 pandemic, and the questionnaire was constructed with a seven-point Likert scale.

### Data collection

This study conducted onsite surveys from 26 September to 30 October 2020. The Macao Light Festival was the survey venue. Participants who were over 18 years of age and had visited at least two event sites were the subjects of the study. This research employed the convenience sampling method. The investigator first asked the respondents about their willingness to complete the survey and continued the survey only if they opted to participate willingly. This research provided respondents who completed the survey with gifts to increase the response rate. A total of 400 questionnaires were distributed, and 338 were returned, including 301 valid questionnaires. Female samples outnumbered male samples in this survey, totaling 187 (62.1%). The target audience for the festival is 18 to 30 years old, and 185 participants (54.6%) were in this range. A total of 219 participants (72.8%) had a bachelor’s degree or higher. These figures are comparable to samples of festival attendees from previous years (Hsu et al., 2021; Wu et al., 2014). However, due to the pandemic, the festival’s audience was primarily composed of Macau residents.

### Data Analysis

The analytical instruments used in this analysis were SPSS 24.0 and Amos 23.0. AMOS is a package of software for performing covariance structure analysis (C-SEM), one of three approaches to structural equation modeling; the other two are partial least squares (PLS) and generalized structured component analysis (GSCA) (Henseler, 2012). Hwang et al. (2010) concluded that C-SEM accurately recovers loadings, parameters, and path coefficients and produces unbiased parameter estimates. Additionally, Henseler (2012) discovered that when compared to C-SEM, GSCA overestimates direct effect in mediation analysis, is partially invalid, and produces inconsistent estimates. For the reasons stated heretofore, this study performed SEM analysis using the C-SEM method. The primary reason for employing AMOS in C-SEM analysis is to take advantage of two significant benefits. To begin, AMOS can be integrated into the widely used SPSS configuration. Second, AMOS is simple to use, with an intuitive graphical communication interface and the ability to estimate SEM models (Babin et al., 2008).

SPSS 24.0 is primarily used to track the accuracy of recovered data. First, we perform descriptive statistics on kurtosis, skewness, and mean. We then perform common method deviation tests, analyze the overall correlation between items in each dimension and perform reliability tests after removing improper items. In the festivalscape section, exploratory factor analysis is used to investigate internal structures, and Amos 23.0 is used for CFA analysis, combination reliability, and convergence validity analysis of the measurement model to determine if any model modifications are needed. Finally, structural equation modeling (SEM) is used to test the overall structure model and the hypotheses. The bootstrap method was employed to test for possible mediation effects.

## Findings

Before data analysis, this study tested the dataset for missing values and outliers and found none. According to kurtosis and skewness metrics, the data are normally distributed and appropriate for further study.

### Common Methods Deviation

To prevent common method variance, the questionnaire layout is based on strategies such as concealing the items’ purposes and randomly arranged questions. In addition, single-factor Harman test is used for remedial mitigation; it is commonly used as a remedial step in this fashion (Podsakoff & Organ, 1986). The test results show that four factors can be extracted and that the explanatory power of the first factor is 44.107%, which does not exceed 50%. Accordingly, there is no serious common method variance in this analysis’s sample data.

### Festivalscape Perception During the Pandemic

This study introduces pandemic prevention initiatives into festival settings. Thus, the methods suggested by Churchill (1979) and Tsaur et al. (2010) were used to measure festivalscape perception. Half the data were randomly selected in this study, and exploratory factor analysis was conducted on the festivalscape. The KMO value of the factor analysis is 0.907, and the Bartlett sphericity test is statistically significant at the 0.000 level, suggesting that these results are suitable for further exploratory factor analysis. In the additional exploratory factor analysis, we used the maximum rotational variance approach for the main component analysis and purified items that met the requirements for a feature value of greater than 1 or a factor loading of greater than 0.5 (Hair et al., 2010). We then used the above criteria to replicate the test on 23 items until all items met them. A total of five items that did not reach the criteria were excluded, leaving 18; the number of factors that were extracted at that time was 3, and the overall total explained variation was 67.406%. The results of this test are shown in Table 3.

**Table 3.** Exploratory Factor Analysis of the Perception of the Festivescape in an Epidemic Situation (n = 150).

Constructs/items	Factor loading	Eigenvalue	Total variance	Interpretation variance	Alpha
EPM		8.630	28.957	28.957	.912
EPM1	0.649				
EPM2	0.708				
EPM3	0.673				
EPM4	0.705				
EPM5	0.833				
EPM6	0.795				
EPM7	0.801				
EPM8	0.601				
EPM9	0.592				
FCT		2.353	23.109	52.065	.899
FS2	0.694				
FS3	0.686				
FS6	0.691				
FS12	0.815				
FS13	0.886				
FS14	0.841				
STF		1.150	15.340	67.406	.924
FS9	0.780				
FS10	0.786				
FS11	0.849				

**Figure 2.**
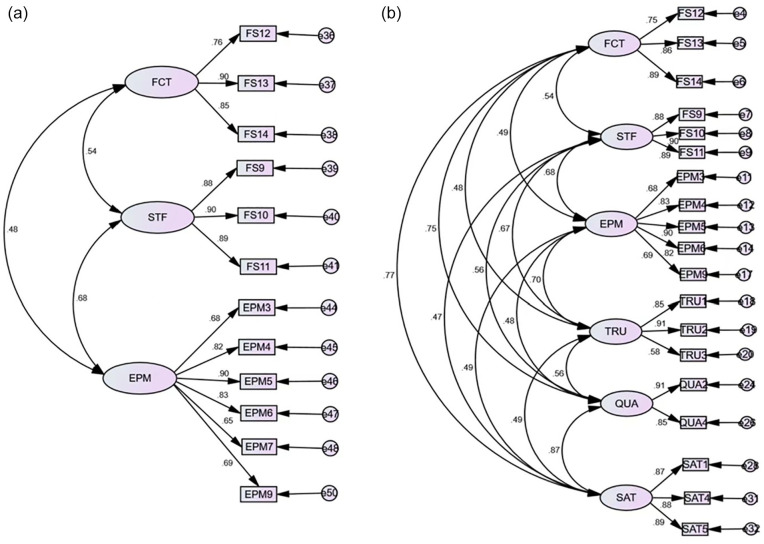
(a) CFA of festivalscape and (b) CFA of the overall measurement model. *Note.* FCT = facility; STF = staff; EPM = epidemic prevention measures; TRU = trust; QUA = experience quality; SAT = satisfaction.

Using the results of this exploratory factor analysis, we conducted a confirmative factor analysis of the three festivalscape dimensions. The festivalscape perception model was refined by using the modification index, and the fitting index of the festivalscape CFA, which met the norm recommended by Hair et al. (2010), was eventually obtained as follows: Δ^2^/df = 1.768, RFI = 0.952, NFI = 0.963, CFI = 0.983, IFI = 0.983, RMSEA = 0.051. Figure 2a illustrates these details.

### Hypothesis Test

This research uses confirmatory factor analysis to assess the reliability and validity of its data (Anderson & Gerbing, 1988). Initially, the AVE square root of experience quality and satisfaction in the original measurement model did not satisfy the criteria of discriminatory validity. Following a revision of the measurement model, based on MI, the fitness index of the measurement model was finally obtained, meeting the standards suggested by Hair et al. (2010), as follows: Δ^2/^df = 1.674, RFI = 0.935, NFI = 0.948, CFI = 0.978, IFI = 0.976, RMSEA = 0.047. CFA analysis of the overall measurement model is shown in Table 4 and Figure 2b.

**Table 4.** The Result of Confirmatory Factor Analysis (n = 301).

Constructs/items	Std. estimate	*t*-Value	Alpha	CR	AVE
**FCT**			.889	0.874	0.699
FS12	0.751				
FS13	0.857	14.994			
FS14	0.893	15.524			
**STF**			.918	0.918	0.788
FS9	0.880				
FS10	0.896	21.500			
FS11	0.888	21.174			
**EPM**			.910	0.890	0.621
EPM3	0.682	11.022			
EPM4	0.827	13.199			
EPM5	0.897	14.146			
EPM6	0.819	13.082			
EPM9	0.694				
**TRU**			.891	0.834	0.635
TRU1	0.853				
TRU2	0.913	18.353			
TRU3	0.584	10.681			
**QUA**			.883	0.876	0.779
QUA2	0.910				
QUA4	0.854	19.741			
**SAT**			.910	0.910	0.772
SAT1	0.869				
SAT4	0.880	20.514			
SAT5	0.886	20.749			

*Note.* FCT = facility; STF = staff; EPM = epidemic prevention measures; TRU = trust; QUA = experience quality; SAT = satisfaction.

The results in Table 4 indicate that the factor loading of the majority of the items is >0.7, the construct reliability (CR) value of each dimension is >0.7, and the average variance extracted (AVE) is >0.5. The results in Table 5 show that the square root of each dimension of the AVE is greater than the Pearson correlation coefficient and of that of the related dimension (the bold entries are the square root of AVE for each construct). The discriminatory validity of each dimension in the scale is therefore generally satisfactory.

**Table 5.** The discriminant validity.

	FCT	STF	EPM	TRU	QUA	SAT
FCT	**0.836**					
STF	0.542	**0.888**				
EPM	0.490	0.675	**0.788**			
TRU	0.480	0.667	0.702	**0.797**		
QUA	0.750	0.565	0.482	0.555	**0.883**	
SAT	0.774	0.472	0.493	0.485	0.868	**0.878**

*Note.* FCT = facility; STF = staff; EPM = epidemic prevention measures; TRU = trust; QUA = experience quality; SAT = satisfaction.

We used the maximum likelihood method to measure the model’s six hypotheses and its subhypotheses. Model fit indicators are generally satisfactory (Hair et al., 2010) and as follows: Δ^2^/df = 1.674, RFI = 0.935, NFI = 0.948, CFI = 0.78, IFI = 0.978, RMSEA = 0.047. Accordingly, all hypotheses are confirmed, although H1a, H2b, H2c, and H4 are nonsignificant (the bold entries are the rejected hypothesis). More detailed results are presented in Table 6.

**Table 6.** Structure Parameter Estimates.

Hypothesis	Path	Std. estimate	S.E.	*t*	*p*	Result
H1a	FCT → TRU	0.090	0.063	1.523	.128	**Rejected**
H1b	STF → TRU	0.320	0.074	4.395	***	Support
H1c	EPM → TRU	0.441	0.086	5.832	***	Support
H2a	FCT → QUA	0.602	0.081	8.976	***	Support
H2b	STF → QUA	0.129	0.088	1.733	.083	**Rejected**
H2c	EPM → QUA	−0.052	0.101	−0.671	.502	**Rejected**
H3a	FCT → SAT	0.281	0.086	3.985	***	Support
H3b	STF → SAT	−0.141	0.072	−2.294	.022	Support
H3c	EPM → SAT	0.149	0.083	2.337	.019	Support
H4	TRU → SAT	−0.042	0.075	−0.642	.521	**Rejected**
H5	QUA → SAT	0.689	0.080	8.618	***	Support
H6	TRU → QUA	0.216	0.089	2.792	.005	Support

*Note.* FCT = facility; STF = staff; EP = epidemic prevention measures; TRU = trust; QUA = experience quality; SAT = satisfaction.

We use the bootstrap (bootstrap = 2000) method to detect the mediating roles of trust and experience quality between festivalscape and satisfaction. We also test a remote mediation role in this study. Data analysis results show that the mediating effect of trust occurs only on the path of **EPM → TRU → QUA → SAT** and is a full mediating effect. The mediation impact of experience quality occurs only on the path of **FCT → QUA → SAT**, which is a partial mediation effect. Accordingly, H7 and H8 are partly supported. The relevant results are shown in Table 7.

**Table 7.** Mediation Effect.

		Percentile 95% CI		Bias-corrected percentile 95% CI		Mediator
Path		Lower	Upper	Lower	Upper	
FCT → SAT	Total effects	0.675	1.053	0.679	1.058	
STF → SAT		−0.181	0.155	−0.179	0.157	
EPM → SAT		0.016	0.430	0.019	0.434	
FCT → SAT	Direct effects	0.102	0.547	0.119	0.560	
STF → SAT		−0.328	−0.001	−0.336	−0.009	
EPM → SAT		−0.010	0.398	−0.003	0.408	
FCT → TRU → SAT	Indirect effects	−0.024	0.010	−0.032	0.007	No
STF → TRU → SAT		−0.063	0.035	−0.069	0.030	No
EPM → TRU → SAT		−0.088	0.043	−0.094	0.039	No
FCT → QUA → SAT		0.292	0.576	0.294	0.581	Yes, Partial
STF → QUA → SAT		−0.037	0.227	−0.028	0.239	No
EPM → QUA → SAT		−0.161	0.108	−0.166	0.102	No
FCT → TRU → QUA → SAT		−0.006	0.043	−0.002	0.051	No
STF → TRU → QUA → SAT		0.002	0.110	0.007	0.120	No
EPM → TRU → QUA → SAT		0.003	0.142	0.011	0.162	Yes, Full

*Note.* FCT = facility; STF = staff; EPM = epidemic prevention measures; TRU = trust; QUA = experience quality; SAT = satisfaction.

## Discussion and Conclusions

One of the contributions of this research is that it incorporates pandemic preventive measures to evaluate festivalscapes while investigating the experiences of festival participants in the context of the COVID-19 pandemic. The relationship between festivalscape and trust demonstrates that staff and pandemic prevention measures are critical components in participant trust building. This conclusion is consistent with Sun et al.’s (2022) conclusion regarding hotels. Festival facilities significantly impact the relationship between festivalscape and experience quality. This reflects Bitner’s (1992) assertion that physical environments can influence customer perception and behavior.

Staff members must therefore not only provide services during a pandemic but also perform pandemic prevention work, as individuals place a higher premium on pandemic prevention measures. According to scholars, hotels can boost customer confidence by implementing pandemic prevention measures, which corroborates Kim and Liu’s (2022) conclusion that social distance facilitates the establishment of customer trust in hotel brands.

As demonstrated by the positive impact of festival trust on experience quality, trust is a prerequisite for consumer experience quality in the context of a pandemic. This conclusion corroborates Biedenbach et al.’s (2019) assertion that amid uncertainty, establishing a trusting relationship between customers and enterprises facilitates improved customer quality perceptions.

However, participants’ trust in festivals did not significantly affect festival satisfaction. The most critical factor in festival satisfaction was still experience quality. According to the two-factor principle of Herzberg (1966), health and safety might be hygiene factors that motivate experience quality amid a pandemic. When a hygiene factor performs well, it does not necessarily generate satisfaction, but when this is not successful, it undoubtedly leads to frustration (Herzberg, 1966). Although this argument was originally based on human resource management, several researchers (i.e., Jensen, 2007; Naumann et al., 2001) have shown that it often applies to customer satisfaction. r Amid the COVID-19 pandemic, festival organizers must thus give priority to health and safety in their resource allocations without neglecting to meet the standards of a tourism experience; otherwise, they will not be able to satisfy festival participants.

Moreover, this research focuses on the ongoing effects of the pandemic and incorporates festival participants’ trust as a critical variable that affects their engagement by discussing the relationship between the festivalscape dimensions of experience quality and festival satisfaction.

This research is one of the few studies to investigate the experience quality and satisfaction of festival participants through empirical research during the COVID-19 pandemic. The findings of this research therefore have significant theoretical and practical value, offering a more comprehensive grasp of the implications of festival quality while providing a better understanding of the experience of festival participants by evaluating quality perception and satisfaction assessment.

### Theoretical Implications

In this research, participant festivalscape perceptions were examined in the context of the COVID-19 pandemic based on an amalgam of normal festivalscape circumstances with specific government requirements of festival organizers. An interesting finding is that the five dimensions of a festivalscape from previous studies (Yang et al., 2011) were reduced to two dimensions after factor analysis, namely, festival facilities and festival staff performances. The multiple pandemic prevention initiatives of festival organizers that were summarized in this study became a single factor in the perception of festival participants. Moreover, this research obtained three dimensions of festivalscapes, namely, festival facilities, festival staff performances, and pandemic prevention measures. These results are very different from the more detailed and diversified festivalscapes suggested by many scholars (Choe et al., 2018; Yang et al., 2011; Yoon et al., 2010). The context of this study is the first large-scale festival that was held in Macau after initially successful pandemic control measures. After half a year of self-isolation, participants had the opportunity to engage in large-scale public leisure events. Thus, the pleasure they experienced may have been sufficient to make them neglect their normal attention to detail (Huang et al., 2012). Indeed, some researchers, such as Assaker and Hallak (2013), have pointed out that “searching for novelty” has a moderating effect on the relationship between satisfaction and a desire to revisit. When people temporarily depart from a stressful life and regain novelty, their happiness will increase significantly. Accordingly, participant perceptions of the festivalscape likely did not focus on specific details.

Regarding the effect of festivalscape perception on the trust, experience quality, and satisfaction of participants, this research also discusses topics that merit elaboration.

For example, festival facilities, festival staff performances and pandemic prevention initiatives all have a significant impact on festival satisfaction, which is consistent with previous research results that indicate a festivalscape should have a significant impact on festival satisfaction (Choe et al., 2018; Mason & Paggiaro, 2012). However, after careful inspection, it was found that the relationship between festival staff performance and festival satisfaction is negative. This apparently contradicts the conclusions of previous research (i.e., Choe et al., 2018; Mason & Paggiaro, 2012), which has shown that festival staff performance has a significant positive effect on festival satisfaction. However, many researchers have investigated this apparently contradictory phenomenon. For example, Chen, Lu et al. (2014) conducted customer satisfaction research on telecommunications services and found that customer satisfaction and dissatisfaction can simultaneously occur. Oliver Richard (1997) developed a three-factor theory, based on two-factor theory, to explain this phenomenon. This three-factor theory separates the attributes of goods or services into three categories: essential attributes, extra psychological attributes, and bivalent attributes. Essential attributes are similar to hygiene factors, that is, a lack of these attributes can lead to dissatisfaction. Extra psychological attributes resemble motivating factors, which can help customers feel positive emotions. Finally, bivalent attributes may occur in various circumstances, leading to either satisfactory or unsatisfactory results. Accordingly, the apparent contradictions in this study’s findings can be explained to an extent. In festivals during normal circumstances, staff performance is more closely linked to quality of service and festival quality by participants. Good staff performance would thus entail positive effects on customer satisfaction. The context of this study, however, was a festival that took place during the COVID-19 pandemic. Staff conduct was thus related to both the standard of service and pandemic prevention, entailing an additional collection of health and safety regulations and procedures not normally found in the hospitality industry (Hu et al., 2021). Furthermore, to prevent infection, workers’ assigned tasks often included measures to safeguard the health and safety of festival participants. Ensuring that festival participants maintain social distance and comply with movement restrictions have therefore become important tasks and even the key job responsibilities of festival staff. Accordingly, staff performance in this study does not effectively reflect festival experience quality and might be negatively associated with festival satisfaction.

In addition, Turner and Krizek (2006) introduced the meaning-centered view of consumer satisfaction that is based on hermeneutics. They assumed that consumers’ perceptions of the meaning of a service should be taken as a starting point to eventually satisfy consumers. Thus, staff behavior in this study has two different meanings. One is that service is an aspect of festival quality; the other is that management of the festival site entails pandemic prevention and control. Under meaning-centered satisfaction theory (Turner & Krizek, 2006), when customers believe that the meaning of a festival service is to maintain onsite pandemic prevention and control, this enables festival participants to build trust in the festival, which affirms the results of this study. Accordingly, this research can also be regarded as a verification of the meaning-centered theory of customer satisfaction in a pandemic context.

While this study has not shown that trust can directly affect the satisfaction of festival participants, the results indicate that pandemic prevention measures in a festival can affect participant satisfaction through a complete mediating effect of trust and quality. This indicates that pandemic prevention strategies can be tailored to encourage participants to immerse themselves in a festival to foster a high-quality experience and eventually generate a higher level of festival satisfaction. This conclusion also supports cue utilization theory; consumers view product information as an indicator of quality (Cox, 1962). A festivalscape thus provides insights into consumer trust and a context for assessing quality evaluation. Thus, festival managers should be mindful of pandemic prevention measures to both protect public health and increase festival satisfaction.

### Management Implications

The COVID-19 pandemic has compelled tourism industries to innovate to provide customers with a safe and dependable experience (Shin et al., 2022). The purpose of this study is to examine the causes and consequences of trust in this context. It therefore provides practical guidance for tourism industries to recover from future pandemics and crises.

First, the findings of this research remind festival managers that regarding festival planning and service management, they should pay attention to bivalent attributes while ensuring that festival participants receive sufficient hygiene care and as many motivating factors as possible. Managers should therefore conduct relevant research to distinguish the bivalent attributes of festival services from a customer’s perspective to carry out festival management in a tailored manner.

Second, managers must work diligently in the postpandemic era to provide a safe and dependable environment for participants. Managers, for example, can control entrant flows by scheduling appointments. Travel providers can also provide tourists with real-time and transparent information about perceived travel threats, such as the health status of frontline employees or the number of visitors to various attractions (Zheng et al., 2022). Simultaneously, every effort should be taken to ensure the safety of a festival’s facilities and equipment, such as safeguarding a venue’s quality and basic lighting facilities and effects (Wong et al., 2015), to further build participant trust.

While managing bivalent attributes, festival organizers should, to facilitate meaning-centered customer satisfaction, concentrate on improving service communications to help customers appreciate the meanings of festival services more positively. Since staff have had to administer the responsibilities of pandemic prevention, their work can instill trust in festival attendees, yet it cannot significantly improve their overall satisfaction. This should remind festival directors that they need to thoroughly explore new methods of staff management to ensure the best possible experiences for participants. For instance, new technologies, contactless services, and other innovations (e.g., ultraviolet light, germ-zapping robots, and so on) (Shin & Kang, 2020) can enhance the effectiveness of pandemic prevention and tourist entertainment.

### Research Limitations and Prospects

This research is an empirical study of large-scale festivals in the context of the COVID-19 pandemic. While it has produced some results, it also has the following limitations:

Because of the pandemic, numerous large festivals were canceled. Thus, only one festival was studied as a case, which could entail a lack of representation. Future research should thus examine the aforementioned relationships in various festival contexts.

Second, the results of this research suggest that three-factor theory can effectively explain festival experiences and participant satisfaction during the pandemic. In the future, when considering business management at festivals, three-factor theory can thus be used as the basis for research.

Third, this study did not account for demographic factors when examining the relationship between festivalscape and trust. Given that demographic characteristics may influence trust, future research should also examine the moderating effects of these variables.
